# Unraveling the Plasma Extracellular Vesicles Proteome: Predictors of Resistance in Immunotherapy of Advanced Melanoma

**DOI:** 10.1002/ijc.70650

**Published:** 2026-07-12

**Authors:** Julian Kött, Glenn Geidel, Carmen M. T. Roeper, Lucija Ačkar, Bente Siebels, Hannah Voß, Hartmut Schlüter, Michaela Schweizer, Kilian Müller, Alessandra Rünger, Tim Zell, Benjamin Deitert, Ali Zeinal‐Abedini, Isabel Heidrich, Stefan W. Schneider, Klaus Pantel, Daniel J. Smit, Christoffer Gebhardt

**Affiliations:** ^1^ Fleur Hiege Center for Skin Cancer Research University Medical Center Hamburg‐Eppendorf Hamburg Germany; ^2^ Department of Dermatology and Venereology University Medical Center Hamburg‐Eppendorf Hamburg Germany; ^3^ Institute of Tumor Biology University Medical Center Hamburg‐Eppendorf Hamburg Germany; ^4^ Section Mass Spectrometry and Proteomics, Center of Diagnostics University Medical Center Hamburg‐Eppendorf Hamburg Germany; ^5^ Center for Molecular Neurobiology Hamburg (ZMNH), University Medical Center Hamburg‐Eppendorf Hamburg Germany

**Keywords:** biomarker, extracellular vesicles, immune checkpoint inhibitor, immunotherapy, mass spectrometry, melanoma, primary resistance, proteomics, secondary resistance

## Abstract

Despite substantial clinical benefit from immune checkpoint inhibitors (ICI), advanced melanoma remains challenging due to frequent treatment resistance. Resistance may be intrinsic (primary) or emerge over time (secondary). Biomarkers predicting distinct resistance phenotypes before therapy are lacking. As key mediators of cellular communication, extracellular vesicles (EVs) represent promising biomarkers. This study aimed to identify baseline EV proteome–derived pathways and biomarkers associated with overall, primary, and secondary resistance to ICI in advanced melanoma and to derive biomarker signatures predictive of progression‐free survival (PFS). EVs were isolated from pretreatment plasma samples of 46 patients with advanced melanoma using size exclusion chromatography and ultracentrifugation. Proteomic profiling was performed by liquid chromatography–mass spectrometry using DIA‐NN. Pathway enrichment and network analyses were conducted using Reactome, Metascape, Cytoscape, and DAVID. Resistance‐associated proteins were integrated into composite biomarker signatures and evaluated for association with PFS. Overall resistance was characterized by enrichment of platelet‐ and complement‐associated pathways. Primary resistance was associated with enhanced Fc gamma receptor (FCGR) signaling and downregulation of KSRP‐associated post‐transcriptional regulatory processes. In contrast, secondary resistance was preceded by distinct baseline EV proteomic patterns involving complement activation and reduced hemostasis‐ and platelet‐related pathways. EV‐derived biomarker signatures for overall, primary, and secondary resistance independently discriminated patients according to PFS. Baseline plasma EV proteomics reveals distinct systemic biological programs associated with different resistance phenotypes to ICI in advanced melanoma. EV‐derived biomarker signatures enable stratification by PFS and warrant validation in larger, multicentric cohorts.

## Abstract (highlights)

What's new?

Immune checkpoint inhibitors (ICIs) improve outcomes in advanced melanoma. Many patients, however, ultimately develop primary or secondary resistance, for which predictive biomarkers remain lacking. Here, pretreatment plasma extracellular vesicle (EV) proteins from advanced melanoma patients were investigated using mass spectrometry and pathway analyses to identify resistance‐associated signatures. Distinct EV proteomic patterns differentiated overall, primary, and secondary resistance, with thromboinflammatory, complement, Fc receptor, and metabolic signatures implicated in resistance mechanisms. Composite EV‐derived biomarker signatures independently predicted progression‐free survival. The findings suggest that baseline plasma EV proteomics may enable early identification of ICI resistance phenotypes and support more personalized immunotherapy strategies.

## Graphical abstract

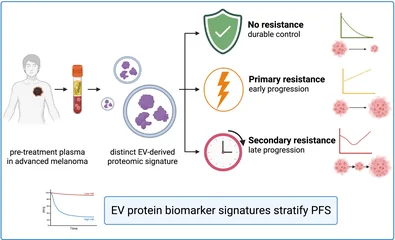

**Abbreviations**
BERT: bidirectional encoder representation from transformers
BH: Benjamini Hochberg
CT: computed tomography
DEP: differentially abundant protein
DIA‐NN: data‐independent acquisition by neural networks
ECOG: Eastern Cooperative Oncology Group
EV: extracellular vesicle
ICI: immune checkpoint inhibition
LDH: lactate dehydrogenase
MRI: magnet‐resonance imaging
NON‐RST: no resistance group
O‐RST: overall resistance group
PBS: phosphate‐buffered saline
PD: progressive Disease
PFS: progression‐free survival
P‐RST: primary resistance group
SD: stable disease
SEC: size exclusion chromatography
SITC: Society for Immunotherapy of Cancer
S‐RST: secondary resistance group
TEM: transmission electron microscopy
UHPLC: ultra‐high‐performance liquid chromatography

## 1 Introduction

Immune‐checkpoint inhibition (ICI) is a standard treatment for advanced melanoma, offering durable remissions in some patients [1]. However, about half of the patients do not benefit due to resistance. The most accepted clinical resistance stratification, defined by Kluger et al. categorizes resistance into primary and secondary forms [2]. Primary resistance reflects the immune system's failure to mount an antitumor response upon initial ICI exposure [2, 3]. It includes patients with progressive disease (PD) as their best response after 6–12 weeks of treatment or those with initial stable disease (SD) followed by PD within 6 months [2]. Proposed mechanisms include ineffective T‐cell priming, defective antigen presentation, and poor tumor infiltration [4, 5, 6]. Secondary resistance occurs as disease progression after an initial response or SD [2, 7]. This may result from adaptive changes such as proteomic or transcriptomic alterations, tumor‐intrinsic adaptations, or clonal selection [5, 8, 9]. The concept of T‐cell exhaustion illustrates how resistance develops as a continuum of functional loss due to persistent antigen exposure. Despite growing research, ICI resistance mechanisms remain poorly understood [4, 7, 10, 11]. Extracellular vesicles (EVs), released by various cells, play a key role in intercellular communication and support tumor progression, angiogenesis, immune evasion, and metastasis [12, 13, 14]. While many studies focus on tumor‐derived EVs, increasing evidence suggests that immune‐ and stromal‐cell‐derived EVs also modulate melanoma progression [15, 16]. Plasma EV cargo analysis may thus provide insights into ICI resistance. However, many clinical trials lack standardized data collection on resistance mechanisms [7]. Recognizing the urgent need for better biomarkers, this study explored the plasma‐derived EV proteome to advance the understanding of ICI resistance in advanced melanoma within a real‐world setting.

## 2 Patients, Materials, and Methods

### 2.1 Patients

This single‐center, retrospective study included 46 AJCC stage III‐IV melanoma patients from the University Skin Cancer Center Hamburg receiving immunotherapy (IT), identified from a prospective biobank. Inclusion criteria required pre‐treatment EDTA plasma and response data based on clinical skin exam, lymph node ultrasound, and imaging (MRI/CT). Resistance classification followed the Society for Immunotherapy of Cancer (SITC) consensus: primary resistance occurred within 6 months of therapy initiation, and secondary resistance occurred later [2]. Demographic and clinical data included sex, age, treatment line, *BRAF* mutation status, melanoma type, tumor thickness, and ulceration status.

### 2.2 EV Isolation

EDTA plasma was centrifuged (1800 × g, then 10,000 × g) to remove cells/debris. Size exclusion chromatography (qEV2 columns, 70 nm pores, IZON) reduced lipoprotein contamination. EVs isolated by ultracentrifugation (100,000 × g, 70 min, 4°C) were resuspended in PBS with protease/phosphatase inhibitors (ROCHE, Germany).

### 2.3 Nanoparticle Tracking Analysis (NTA) and Total Protein Quantification

EVs (1:100 in PBS) were analyzed using NanoSight NS14C (5 × 30 s videos per sample). Particle concentrations were adjusted for dilution. Protein concentrations were measured with the Qubit assay kit. Transmission electron microscopy (TEM) imaging was conducted according to the protocol by Théry et al. using a JEM‐2100Plus microscope at 200 kV with an XAROSA CMOS camera [17].

### 2.4 Sample Preparation for and Liquid Chromatography‐Mass Spectrometry (LC–MS/MS)

50 μL of EV samples were dissolved in 50 μL 100 mM triethyl ammonium bicarbonate (TEAB) and 1% w/v sodium deoxycholate (SDC) buffer, boiled at 95°C for 5 min and sonicated with a probe sonicator. EV samples were dried in a vacuum concentrator and dissolved in 25 μL MS grade water. The samples were then divided into three batches and pipetted into 96‐well LoBind plates (Eppendorf, Hamburg, Germany) placed on an Andrew+ Pipetting Robot (Waters, Milford, USA), which was used to execute all following steps. Using the robot, disulfide bonds were first reduced in 10 mM dithiothreitol for 30 min at 56°C while shaking at 800 rpm and alkylated in presence of 20 mM iodoacetamide for 30 min at 37°C, again while shaking at 800 rpm. Then, carboxylate modified magnetic E3 and E7 speed beads (Cytvia Sera‐Mag, Marlborough, USA) at 1:1 ratio in LC–MS grade water were added in a 10:1 (beads/protein) ratio to each sample, following the single‐pot, solid‐phase enhanced sample preparation (SP3)‐protocol workflow [18]. To bind the proteins to the beads, acetonitrile (ACN) concentration was raised to 50%. Subsequently, samples were shaken at 600 rpm for 18 min at room temperature. Magnetic beads were magnetized, and the supernatant was removed. Magnetic beads were further washed two times with 80% Ethanol (EtOH) and then two times with 100% ACN. After resuspension in 100 mM AmBiCa, digestion with trypsin was performed (sequencing grade, Promega) at 1:100 (enzyme: protein) ratio at 37°C overnight while shaking at 500 rpm. The next day, trifluoroacetic acid (TFA) was added to a final concentration of 1% to inactivate trypsin. The samples were then shaken at 500 rpm for 5 min at room temperature. Finally, beads were magnetized, and the supernatant containing tryptic peptides was transferred into a new 96‐well LoBind plate, ready for subsequent LC–MS/MS analysis.

### 2.5 LC–MS/MS Measurement

Chromatographic separation of tryptic peptides was achieved with a two‐buffer system (buffer A: 0.1% FA in H2O, buffer B: 0.1% FA (Fisher Chemical, A117‐50) in 80% ACN) on a UHPLC (VanquishTM neo UHPLC system, Thermo Fisher). Attached to the UHPLC was a peptide trap (300 μm × mm, C18, PepMap Neo Trap Cartridge, Thermo Fisher, 174,500) for online desalting and purification, followed by a 25 cm C18 reversed‐phase column (75 μm × 250 mm, 130 Å pore size, 1.7 μm particle size, peptide BEH C18, nanoEase, Waters, 186,008,795). Peptides were separated using a 55 min method with linearly increasing ACN concentration from 2.5% to 37.5% buffer B over 45 min. MS/MS measurements were performed on a quadrupole‐orbitrap hybrid mass spectrometer (Exploris 480, Thermo Fisher Scientific). Eluting peptides were ionized using a nano‐electrospray ionization source (nano‐ESI) with a spray voltage of 1800 and analyzed in data independent acquisition (DIA) mode. For each MS1 scan, ions were accumulated for a maximum of 240 milliseconds or until a charge density of 3 × 106 ions (AGC Target) was reached. Fourier‐transformation based mass analysis of the data from the orbitrap mass analyzer was performed covering a mass range of m/z 400–1200 with a resolution of 120,000 at m/z 200. Within a precursor mass range of m/z 380–980 fragmentation in DIA‐mode with m/z 12 isolation windows and m/z 1 window overlaps was performed. Fragmentation was performed at normalized collision energy of 28% using higher energy collisional dissociation (HCD). An AGC target of 2 × 106 ions or a maximum of 54 ms was set. Orbitrap resolution was set to 30,000.

### 2.6 Data Analysis and Visualization

LC–MS/MS data were processed in Proteome Discoverer (Version 3.1.0.638) with the CHIMERYS algorithm [19] and searched against a reviewed *homo sapiens* database (November 2023). The oxidation of methionine was allowed as variable modifications, carbamidomethylation of cysteine was set as fixed modification. A maximum number of one missing tryptic cleavage was set. Peptides between 7 and 30 amino acids were considered. A strict cutoff (FDR < 0.01) was set for peptide identification. Normalization was performed based on the sample median protein abundance. Obtained protein abundances were log2‐transformed. Batch effect correction was performed for the three preparation batches with the BERT algorithm [19] with standard settings. Further statistical analysis was conducted using RStudio (R v4.3.2) with survival and ggplot2 packages, and Python using pandas DataFrame. Partial Least Squares Discriminant Analysis (PLS‐DA) was applied to identify multivariate protein abundance patterns that distinguish non‐resistant from primary‐ and secondary‐resistant patients. Univariable logistic regression analysis included z‐standardization of age and protein covariates to ensure comparability in scaling off non‐binary covariates. *p* values of differentially abundant proteins (DEPs) were adjusted for multiple testing using the Benjamini‐Hochberg (BH) method. No proteins reached significance at an FDR threshold of 0.05 [20, 21]. For exploratory analysis and biological interpretation, we selected proteins with nominal *p* values < 0.05 and a log2‐fold change ≥ Δ1 for further analysis. Pathway and protein interaction analyses of DEPs were conducted via Reactome, Metascape, and Cytoscape [22, 23, 24, 25]. Pathway enrichment used Reactome, KEGG, and WikiPathways, while protein–protein interaction (PPI) networks were analyzed in STRING and visualized in Cytoscape via Metascape [26]. Biological pathway representations were generated using Reactome‐based Voronoi diagrams. In Kaplan–Meier analysis, progression‐free survival (PFS) was defined as the time from initiation of ICI to radiologically or clinically confirmed disease progression or death from any cause. Biomarker signatures were calculated based on the direction and strength of association of individual proteins and dichotomized at the median.

## 3 Results

### 3.1 Patient Characteristics

A cohort of 46 AJCC stage III‐IV metastatic melanoma patients, for whom pre‐treatment plasma was available in the institutional biobank, was identified. Among 31 patients developing overall treatment resistance (O‐RST), 20 were considered primarily resistant (P‐RST) (within 6 months) and 11 secondarily resistant (S‐RST) according to the SITC definition (Table S1) [2]. The median follow‐up was about 27.8 months. Non‐resistant patients (NON‐RST), that is, those with complete response (CR), partial response (PR), or SD for more than 6 months without further evidence of PD, were followed up for a median of 49.5 months (Table S1). Melanoma subtypes were not deliberately excluded and were evenly distributed across the cohort groups. So did Eastern Cooperative Oncology Group Status (ECOG), *BRAF* mutation status, and other parameters, such as S100‐ and LDH‐level increases upon treatment initiation. Most patients received the combination of ipilimumab and nivolumab, which was introduced as a first‐line treatment (Table S1).

### 3.2 EV Isolation and Overall Data Characterization

Nanoparticle tracking analysis (NTA) confirmed similar EV size distributions across groups (Figure S1A–C). The central single peak was observed at approximately 130 nm, consistent with the typical size distribution of EVs. TEM confirmed EV presence and purity (Figure S1D,E). The proteomic dataset was aligned with the clinical dataset. Venn analysis identified 2108 EV proteins: 1734 in non‐resistant, 1686 in primary resistant, and 1710 in secondary resistant patients (Figure 1A). Unique proteins included 153 in non‐resistant patients, 141 in primary resistant patients, 122 in secondary resistant patients, and 1330 proteins (63.1%) were shared (Figure 1A). In a two‐group PLS‐DA, patients with resistance overall showed distinct baseline proteomic signatures compared to non‐resistant patients (Figure 1B). A three‐group PLS‐DA demonstrated separable signatures for non‐resistant, primary resistant, and secondary resistant patients, suggesting that resistance phenotypes are associated with discrete baseline protein abundance patterns (Figure 1C). To explore protein differences between non‐resistant and resistant patients, volcano plot analysis was performed (Figure 1D). After correction for multiple testing, no proteins remained statistically significant. Hence, we further used proteins with nominal significance and a log2‐fold change of ≥ Δ1 to identify candidate markers for biological pathways and treatment response. Among these, 16 candidate proteins were significantly lower abundant, and 13 were significantly higher abundant in non‐resistant patients (Table S2).

**FIGURE 1:**
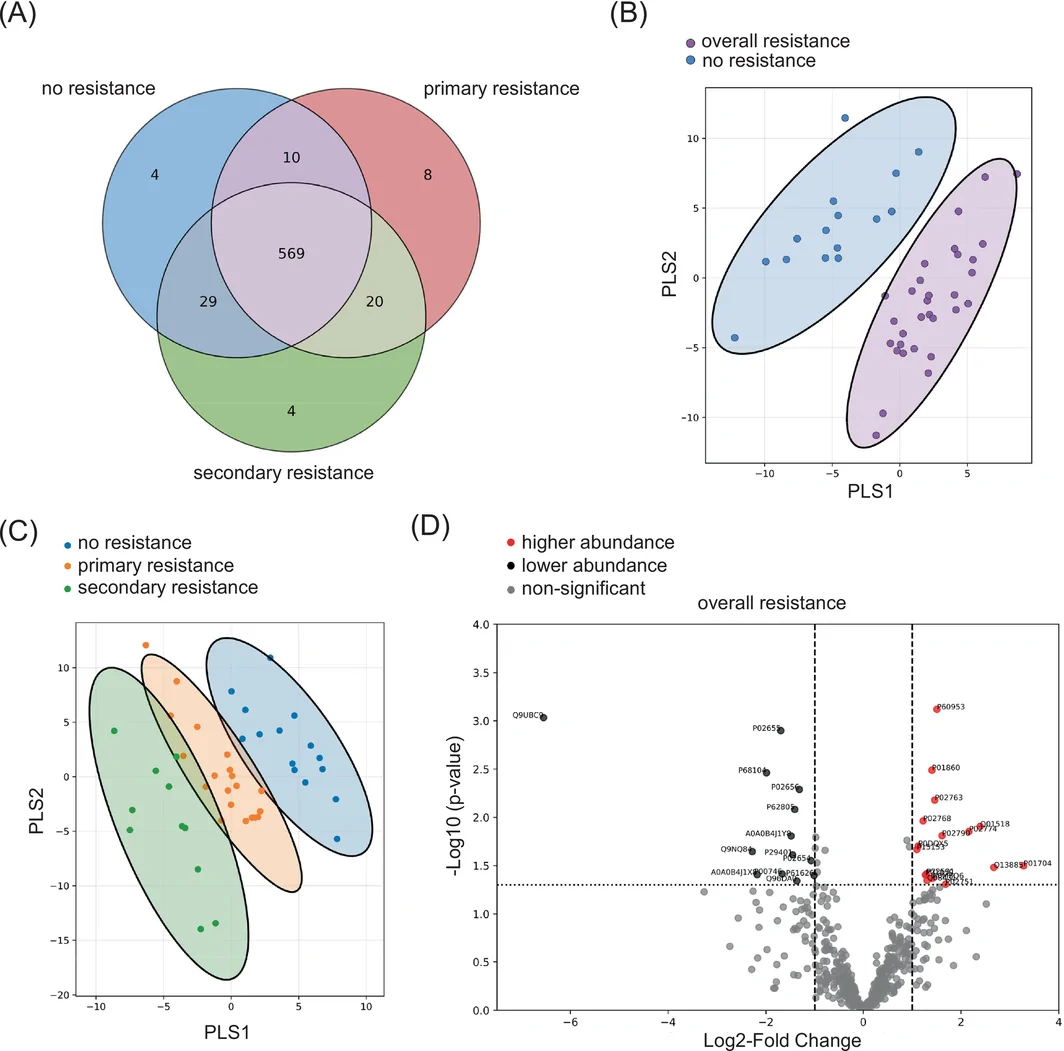
EV characterization and overall proteomic exploration. (A) Venn diagram comparing proteins detected in the no resistance, primary resistance, and secondary resistance groups. (B, C) Partial least‐squares discriminant analysis plots of (B) no resistance and overall resistance groups, and (C) no resistance, primary resistance, and secondary resistance groups. (D) Volcano plot depicting in red proteins with higher abundance and in black proteins with lower abundance in the overall resistance group compared to the non‐resistance group on a scale of −log10 *p* value and log2‐Fold Change. Dotted horizontal lines indicate the level of significance at *p* value < 0.05 (horizontal line), and log2‐fold change > 1.0 and < −1.0 (vertical lines), respectively.

### 3.3 Identification of Overall Resistance Mechanisms and Extraction of Predictive Biomarkers

To explore curated biological pathways involved in overall resistance, we performed Metascape analysis of the protein sets from the non‐resistance and overall resistance group. Based on proteins showing nominal significance, pathway enrichment analysis identified five significantly enriched pathway clusters in the overall resistant group after FDR correction, including platelet activation, signaling, and aggregation (Table S3). Within these enriched pathways, the six candidate proteins cell division cycle 42 (CDC42), orosomucoid 1 (ORM), albumin (ALB), CAP1, hemopexin (HPX), and Rac family‐small GTPase 2 (RAC2) were identified as key contributors. Hierarchical clustering based on Z‐score normalized abundance of these proteins indicated a clear separation of non‐resistant and overall resistant patients, with both primary and secondary resistance phenotypes displaying increased baseline abundance (Figure 2A). To further contextualize the biological relevance of the enriched pathways and to explore functional interconnections between resistance‐associated proteins, we performed a network‐based enrichment analysis using Cytoscape. Pathway annotation across Reactome, KEGG, and WikiPathways highlighted pathways linked to immune modulation, inflammation, hemostasis, and platelet activation as central biological processes associated with overall resistance (Figure 2B).

**FIGURE 2:**
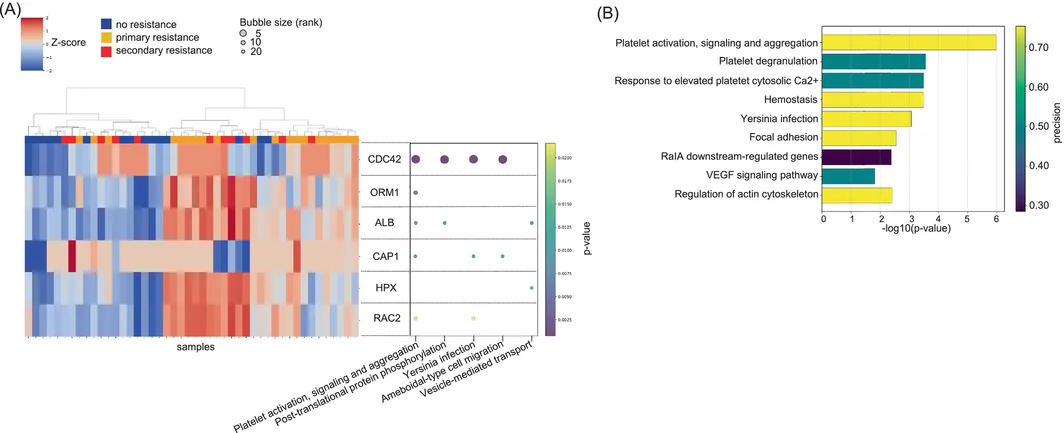
Biological pathway and biomarker analysis in overall resistant patients. (A) Clustered hierarchical heatmap of the top 5 Metascape‐enriched pathway clusters, Z‐score normalized, alongside a bubble plot of the top 5 Metascape‐enriched pathway clusters. Bubble size displays the rank of differentially abundant protein after Benjamini‐Hochberg correction. Bubble color is based on the *p* values of the top six proteins with higher abundance in the overall resistance group, identified by Metascape clustering. The heatmap and bubble plot are interconnected via the depicted protein names. The resistance group is coded in an extra line above protein data, with no resistance group in blue, primary resistance in yellow, and secondary resistance in red. (B) Bar plot displaying the Cytoscape enriched Reactome, KEGG, and WikiPathways in overall resistant patients alongside −log10 *p* value at the significance level of *p* < 0.05. Color‐coded precision mapping according to the number of intersecting genes versus the total number of genes.

To evaluate the clinical relevance of resistance‐associated proteins, we performed uni‐ and multivariable logistic regression analyses on a predefined set of previously identified candidate biomarkers. This set comprised the five most strongly up‐ and downregulated proteins and included proteins contributing to the top enriched pathway clusters identified by Metascape (Table S4). In multivariable models, elevated baseline levels of IGHG3 (OR 2.43, 95% CI 1.14–5.15, *p* = 0.021) and IGKC (OR 2.03, 95% CI 1.05–3.91, *p* = 0.034) were independently associated with increased odds of overall treatment resistance (Table S4). In contrast, higher levels of APOC2 (OR 0.30, 95% CI 0.13–0.71, *p* = 0.007), APOC3 (OR 0.16, 95% CI 0.04–0.69, *p* = 0.024), and HIST1H4A (OR 0.43, 95% CI 0.19–0.98, *p* = 0.045) were independently associated with decreased odds of overall resistance towards ICI (Table S4).

### 3.4 Characterization of Primary Resistance Mechanisms and Extraction of Potentially Predictive Biomarkers

Based on the findings on overall resistance, we next focused on identifying molecular mechanisms and biomarkers associated with primary resistance, which reflects intrinsic treatment resistance to ICI. To identify significant proteins that differ between primary‐resistant and non‐resistant patients, we performed volcano plot analysis (Figure 3A). As no proteins reached statistical significance after multiple testing correction, proteins with nominal significance (*p* < 0.05) and a log2‐fold change of ≥ Δ1 were selected for exploratory downstream analyses (Table S5).

**FIGURE 3:**
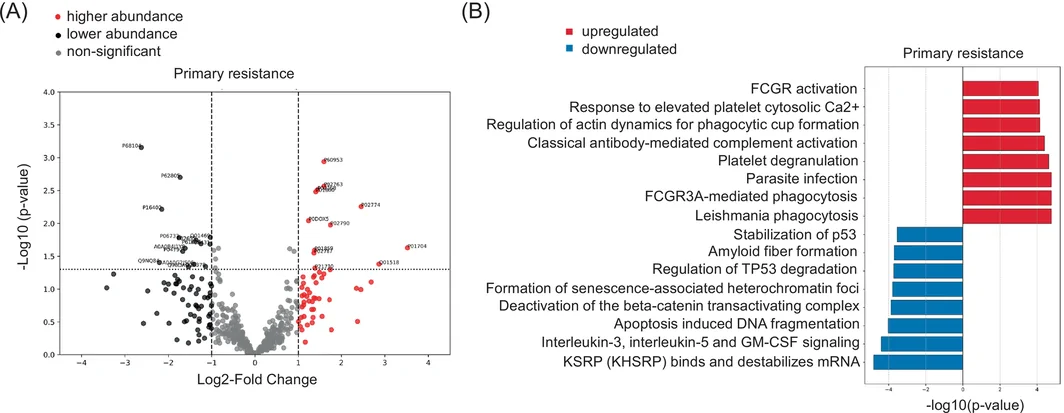
Biological pathway analysis and biomarker identification in primary resistance. (A) Volcano plots depicting in red proteins with higher and in black proteins with lower abundance in primary resistance group compared to the non‐resistance group on a scale of −log10 *p* value and log2‐Fold change. Level of significance at *p* value < 0.05 (horizontal line) and log2‐fold change > 1.0 and < −1.0 (vertical lines), respectively. (B) Bar plot depicting in red top 8 upregulated and in blue top 8 downregulated Reactome pathways in primary resistance group.

To visualize enriched biological processes in a pathway‐centric manner, Reactome‐based Voronoi diagrams were generated. This approach revealed significant upregulation of processes related to neutrophil degranulation, complement cascade activation, and Fc gamma receptor (FCGR) signaling in patients with primary resistance (Figure S2A). In contrast, pathways associated with KH‐type splicing regulatory protein (KRSP)‐mediated mRNA destabilization were downregulated (Figure S2B). Reactome pathway enrichment analysis further confirmed immune‐related and platelet‐associated pathways, including complement activation and platelet degranulation, among the top upregulated processes in the primary resistant group (Figure 3B).

To assess the potential clinical relevance of these findings, multivariable logistic regression analysis was performed on top‐ranked candidate proteins. Elevated baseline levels of IGHG3 (OR 4.19, 95% CI 1.22–14.37, *p* = 0.022) and IGKC (OR 6.00, 95% CI 1.30–27.71, *p* = 0.022) were independently associated with increased odds of primary resistance, whereas higher APOC2 levels (OR 0.38, 95% CI 0.16–0.86, *p* = 0.027) were associated with decreased odds of primary resistance to treatment (Table S6).

### 3.5 Characterization of Secondary Resistance Mechanisms and Extraction of Potentially Predictive Biomarkers

Next, we investigated molecular pathways and candidate biomarkers associated with secondary resistance, potentially reflecting resistance that emerges after an initial exposure to and response to ICI. As in the previous analysis, DEPs comparing secondary resistant to non resistant patients were not consistently significant after multiple testing correction (Figure 4A; Supplementary Table S7). We therefore selected proteins with nominal significance (*p* < 0.05) and a log2‐fold change of ≥ Δ1 for exploratory downstream analyses. Reactome‐based Voronoi projections of upregulated pathways highlighted a pronounced enrichment of innate immune and myeloid‐associated processes in the secondary resistance group (Figure S3A). Prominent pathways included neutrophil degranulation, events associated with phagocytolytic activity of polymorphonuclear cells, and inflammasome‐related signaling. In addition, pathways involved in antigen processing via ubiquitination and proteasome‐mediated degradation, including ER‐phagosome and cross‐presentation, were prominently represented. In contrast, Voronoi projections of downregulated pathways were dominated by hemostasis‐ and platelet‐associated biological processes (Figure S3B). Consistent with these projections, Reactome pathway ranking identified innate immune and hemostatic effector pathways among the most significantly upregulated processes in the secondary resistance group (Figure 4B). The most significantly downregulated pathways were dominated by lipid and lipoprotein metabolism.

**FIGURE 4:**
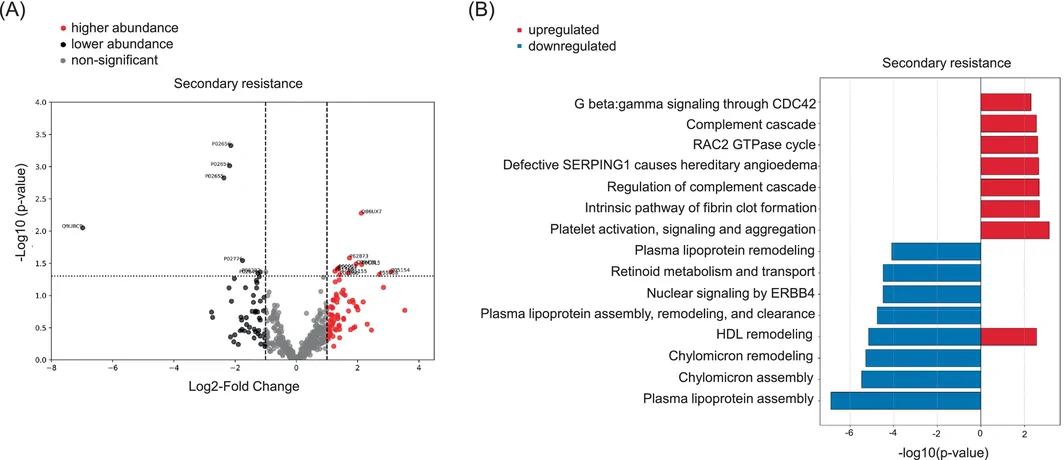
Biological pathway analysis and biomarker identification in secondary resistance. (A) Volcano plots depicting in red proteins with higher and in black proteins with lower abundance in the secondary resistance group compared to the non‐resistance group on a scale of −log10 *p* value and log2‐Fold change. Level of significance at *p* value < 0.05 (horizontal line) and log2‐fold change > 1.0 and < −1.0 (vertical lines), respectively. (B) Bar plot depicting in red the top 8 upregulated and in blue the top 8 downregulated Reactome pathways in the secondary resistance group.

To assess the potential clinical relevance of individual proteins associated with secondary resistance, uni‐ and multivariable logistic regression analyses were performed. Elevated baseline levels of APOC1 (OR 0.23, 95% CI 0.05–0.59, *p* = 0.047) were independently associated with decreased odds of secondary resistance (Table S6). Other candidate proteins did not retain independent significance in multivariable models or could not be reliably evaluated due to limited subgroup sizes.

### 3.6 Computation of Predictive Biomarker Signatures

We next computed composite biomarker signatures to stratify patients according to progression risk, based on previous comparative analyses between the groups. Kaplan–Meier survival analyses were performed to evaluate PFS according to biomarker signature scores (Figure 5A–C). For overall resistance, low scores were significantly associated with a shorter PFS compared to non‐resistant patients (median PFS 6.4 months O‐RST vs. not reached NON‐RST). In contrast, for primary resistance, higher biomarker signature scores were associated with prolonged PFS (median PFS 4.6 months P‐RST vs. not reached NON‐RST). In multivariable Cox proportional hazards analysis, both biomarker signatures remained independently associated with disease progression after adjustment for clinical covariates, predicting progression in the overall resistance (HR 4.39, 95% CI 1.73–11.14, *p* = 0.002) and primary resistance (HR 5.74, 95% CI 1.66–19.84, *p* = 0.005) groups (Table S8).

**FIGURE 5:**
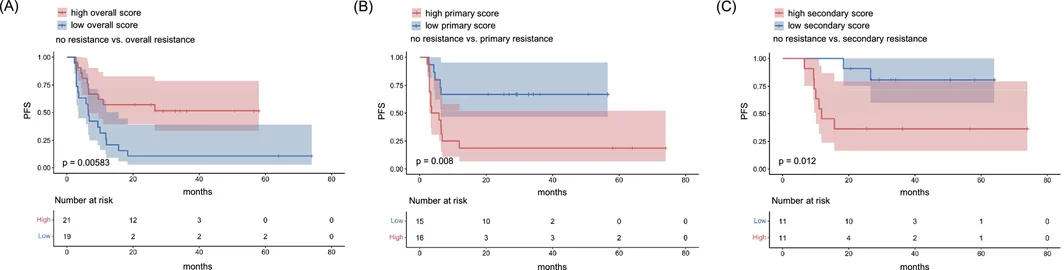
Discrimination of progression‐free survival with biomarker signatures. (A–C) Kaplan–Meier analysis of progression‐free survival (PFS) applying computed biomarker signatures of overall resistance, including IGHG3, APOC2, APOC3, HIST1H4A, IGKC, and APOC1 (A); primary resistance, including IGHG3, IGKC, and APOC2 (B); and secondary resistance, including APOC1 only (C). The colored area depicts confidence interval of 95%. Log‐rank test showing significance at *p* value < 0.05.

APOC1 was previously identified as the only protein independently associated with secondary resistance. Accordingly, the biomarker signature for secondary resistant patients consisted of this single marker (Figure 5C). Low APOC1‐based signature scores were associated with prolonged PFS in Kaplan–Meier analysis (median PFS 11.6 months S‐RST vs. not reached NON‐RST). Multivariable Cox regression confirmed an independent association with disease progression (HR 8.76, 95% CI 137–55.66, *p* = 0.021), although confidence intervals were wide due to limited subgroup size (Table S8).

## 4 Discussion

This study aimed to identify EV proteome‐based pathways and baseline biomarkers of ICI resistance in advanced melanoma. This study systematically characterizes EV‐derived proteomic signatures of overall, primary, and secondary resistance and explores the integration of these findings into composite biomarker signatures potentially predictive of PFS.

O‐RST group was characterized by enrichment of complement activation and platelet‐associated pathways, indicating a systemic thromboinflammatory state. Complement signaling has been linked to tumor‐promoting inflammation through the C5a/C5aR1 axis, promoting myeloid‐derived suppressor cell recruitment and immune evasion [27, 28]. Consistent with this, complement‐mediated neutrophil activation has been shown to impair vascular integrity and enhance melanoma dissemination [29]. In parallel, increased platelet activation and aggregation pathways were observed, aligning with reports linking thromboinflammatory processes and NETosis to unfavorable outcomes in ICI‐treated melanoma patients [30]. The concurrent enrichment of complement and platelet‐associated pathways supports the notion of coordinated interactions between innate immune and hemostatic systems rather than isolated pathway activation, a concept increasingly recognized across cancer entities, including melanoma [31, 32, 33].

Beyond pathway enrichment, several individual proteins were independently associated with overall resistance in univariable and multivariable analyses, including immunoglobulin‐related proteins and apolipoproteins. At first glance, the prominence of immunoglobulin chains and lipid‐associated proteins in EV‐based analyses may raise concerns regarding technical confounding or plasma contamination. However, accumulating evidence suggests that both immunoglobulins and apolipoproteins are actively incorporated into EVs and can reflect biologically relevant immune and metabolic states rather than mere technical artifacts [34, 35, 36]. In the O‐RST group, the association of immunoglobulin‐related proteins may reflect enhanced antibody‐mediated immune activity. Apolipoproteins may indicate altered lipid transport and inflammatory metabolism, processes increasingly implicated in shaping immune responses and tumor progression [37, 38]. Importantly, EV isolation by size‐exclusion chromatography reduces, but does not entirely eliminate, co‐isolation of lipid‐associated particles, necessitating cautious biological interpretation of these signals.

Within this thromboinflammatory background, the P‐RST group was associated with a more specific immune configuration characterized by enrichment of FCGR signaling, neutrophil‐associated processes, and platelet‐related biological programs. In contrast to O‐RST, FCGR signaling was especially upregulated in P‐RST, suggesting a baseline immune state dominated by Fc‐mediated effector mechanisms. FCGR signaling has been proposed to function as an immunoregulatory checkpoint, modulating antibody‐mediated immune responses and potentially conferring antibody drug resistance [39, 40, 41]. When these pathways are upregulated at baseline, immune activity may be biased toward innate effector and clearance pathways, thereby potentially limiting the capacity to mount effective T cell‐mediated antitumor responses upon ICI exposure.

Notably, the P‐RST group showed downregulation of KSRP‐associated pathways, including mRNA destabilization processes. KSRP plays a central role in post‐transcriptional regulation of immune‐related transcripts and has been implicated in controlling inflammatory gene expression, cytokine turnover, and immune cell activation [42, 43, 44]. Reduced KSRP‐associated pathways may therefore contribute to dysregulated immune signaling and impaired immune adaptability at baseline.

The S‐RST group exhibited a distinct biological pattern, with enrichment of antigen‐processing‐related pathways, including ubiquitination and proteasome‐mediated degradation, alongside complement cascade activation and innate immune signaling. These findings could delineate a latent antigen‐handling and innate effector program at baseline that may predispose tumors to later immune escape. At the same time, network‐based analyses demonstrated downregulation of hemostasis‐ and platelet‐associated pathways, including platelet degranulation and GPVI‐mediated activation cascades. This contrasts with the platelet‐dominated signatures observed in the other groups and suggests that a different balance of vascular and immune interactions may precede secondary resistance. Interestingly, Reactome‐based ranking further indicated both up‐ and downregulation of HDL remodeling, plasma lipoprotein assembly, and clearance. While this bi‐directional regulatory signal may initially seem contradictory, it may reflect the aggregation of functionally distinct subpathways within broader pathway categories rather than actual biological inconsistency. Alternatively, it could indicate heterogeneity in EV origin, with contributions from platelet‐derived, hepatocyte‐derived, and immune cell‐derived vesicles differing between patients.

The presence of lipid‐ and retinoid‐related pathways, as well as nuclear signaling by ERBB4, further underscores the involvement of metabolic and signaling processes in shaping baseline resistance phenotypes. Although co‐isolation of lipoproteins remains a technical consideration in plasma EV studies, increasing evidence supports a functional interplay between lipid metabolism, EV biology, and immune regulation [45, 46]. Consequently, these findings may be best interpreted as biological signals reflecting systemic immune‐metabolic states rather than technical contamination alone, albeit requiring cautious validation in independent cohorts.

At the protein level, APOC1 emerged as the only marker independently associated with secondary resistance in multivariable analysis. APOC1 has been implicated in lipid metabolism and immune modulation and has been linked to tumor progression and immune evasion in different cancer entities [38, 47, 48]. Its association with the S‐RST group could suggest that baseline alterations in lipid‐associated immune regulation may predispose patients to later loss of ICI efficacy, even in the absence of resistance at treatment initiation. However, because baseline plasma was analyzed, the dynamics of the EV proteome could not be examined to explore this mechanism further. They should thus be included in future studies of S‐RST melanoma patient cohorts.

Beyond individual pathways and proteins, composite biomarker signatures derived from resistance‐associated markers for O‐RST and P‐RST independently predicted disease progression. These findings support that baseline EV proteomic signatures may capture systemic biological information that extends beyond conventional prognostic factors. For S‐RST, the identification of a single‐protein signature may underscore the possibility of a scarcity of factors identifiable at baseline, independently indicating a later‐developing adaptive resistance.

Several limitations warrant consideration. All analyses were performed on baseline samples, precluding direct assessment of dynamic or therapy‐induced biological changes. Consequently, the identified signatures should be interpreted as pre‐existing molecular configurations or traces rather than evolving resistance mechanisms. In addition, no individual proteins remained statistically significant after correction for multiple testing, and pathway analyses were conducted in an exploratory framework aimed at hypothesis generation. The monocentric design and moderate cohort size further limit generalizability, and technical aspects of plasma EV isolation warrant cautious interpretation of lipid‐associated signals. Limited follow‐up may constrain the interpretability of overall survival outcomes. Distant metastasis‐free survival was not evaluated, as a substantial proportion of patients presented with metastatic disease at baseline and distant progression was not captured separately. Additionally, the identified proteins should not be viewed as definitive markers but rather as preliminary candidates that may reflect underlying biological processes. Their inclusion in pathway and enrichment analyses is intended to support hypothesis generation and guide future validation efforts. The computed biomarker signatures showed strong discrimination, hazard ratios (HRs) and confidence intervals (CIs) were broad, especially for P‐RST and S‐RST groups. Small sample sizes may have contributed to variability, leading to less precise HR estimates. Although a strong predictive effect was observed, proteins included in the signature were sometimes measured in only a subset of patients, further increasing variability. Nevertheless, the consistency of pathway‐level patterns and the independence of biomarker signatures in multivariable models support the biological relevance of the observed findings and justify further validation in larger, longitudinal cohorts.

Together, this study demonstrates that baseline plasma extracellular vesicle proteomics captures biologically meaningful systemic programs associated with resistance to immune checkpoint inhibition in advanced melanoma. Thromboinflammatory and Fc‐dependent innate immune signatures characterize overall and primary resistance. In contrast, secondary resistance is preceded by distinct baseline patterns involving antigen processing, complement activation, metabolic pathways, and altered platelet‐related signaling. Integration of EV‐derived proteins into biomarker signatures enables stratification of patients by PFS, highlighting the potential of EV‐based proteomics as a liquid biopsy for early resistance assessment. While exploratory, these findings provide a mechanistic framework for future validation studies and underscore the relevance of baseline systemic immune–metabolic states in shaping immunotherapy outcomes.

## Author Contributions

**Julian Kött:** conceptualization, funding acquisition, data curation, formal analysis, methodology, project administration, software, supervision, visualization, writing – original draft, investigation, validation, writing – review and editing. **Glenn Geidel:** conceptualization, funding acquisition, data curation, formal analysis, methodology, project administration, software, supervision, visualization, writing – original draft, writing – review and editing, investigation, validation. **Carmen M. T. Roeper:** data curation, formal analysis, methodology, visualization, writing – original draft, writing – review and editing, investigation, validation. **Lucija Ačkar:** data curation, methodology, writing – review and editing. **Bente Siebels:** data curation, methodology, software, writing – review and editing, investigation, validation. **Hannah Voß:** data curation, methodology, writing – review and editing, investigation. **Hartmut Schlüter:** resources, writing – review and editing. **Michaela Schweizer:** data curation, methodology, writing – review and editing. **Kilian Müller:** data curation, methodology, writing – review and editing, investigation. **Alessandra Rünger:** writing – review and editing. **Tim Zell:** writing – review and editing. **Benjamin Deitert:** writing – review and editing. **Ali Zeinal‐Abedini:** writing – review and editing. **Isabel Heidrich:** writing – review and editing. **Stefan W. Schneider:** resources, writing – review and editing. **Klaus Pantel:** conceptualization, resources, writing – review and editing. **Daniel J. Smit:** data curation, methodology, resources, writing – review and editing. **Christoffer Gebhardt:** conceptualization, project administration, resources, supervision, writing – review and editing.

## Funding

This research was funded by a grant from the Hiege‐Stiftung—die Deutsche Hautkrebsstiftung to G.G. and J.K. This study was supported by grants from the Deutsche Forschungsgemeinschaft (247354600, 247377969, 426788273, 518551069, 516868494) to H.S., B.S., and K.M.

## Ethics Statement

This study was conducted in accordance with the Declaration of Helsinki and approved by the Ethics Committee of the Hamburger Ärztekammer (approval number: PV5392). All subjects provided written informed consent to participate in the study and to be included in the institutional biobank.

## Conflicts of Interest

G.G. has received honoraria from Almirall Hermal, BMS, SUN‐Pharma, Janssen‐Cilag, and Mylan, outside the submitted work. G.G. has received travel expenses from MSD, Pierre Fabre, and SUN Pharma, outside the scope of the submitted work. G.G. has received grants from Regeneron Pharmaceuticals and Delcath Systems, outside the submitted work. G.G. is a member of the advisory board of Regeneron. G.G. is an unpaid board member of SCOPE. J.K. has received honoraria from Bristol‐Myers Squibb and Sanofi Genzyme outside the submitted work. J.K. has received travel support from SUN Pharma and Pierre Fabre outside the submitted work. B.S. has received grants from the Deutsche Forschungsgemeinschaft (DFG, German Research Foundation, 518551069). H.S. has received grants from the Deutsche Forschungsgemeinschaft (247354600, 247377969, 426788273, 518551069, 516868494). C.G. is a member of the advisory board of BMS, Immunocore, MSD, Novartis, Pierre‐Fabre, Sanofi, SUN Pharma, Sysmex. C.G. has received honoraria from BMS, GSK, Immunocore, MSD, Novartis, Pierre‐Fabre, Sanofi, SUN‐Pharma, Sysmex, outside the submitted work. C.G. has received travel expenses by BMS, Pierre‐Fabre, SUN Pharma, outside the submitted work. C.G. is a board member of the DeCOG (ADO), unpaid; C.G. is a board member of the Hiege Stiftung, unpaid. C.G. is a board member of the Roggenbuck‐Stiftung, unpaid. C.G. is founder of Dermagnostix and Dermagnostix R&D. The other authors declare no conflicts of interest.

## Supporting information


**Table S1:** Patient characteristics. Clinical characteristics of the advanced melanoma patient cohort showed no resistance, primary resistance, and secondary resistance groups upon immune checkpoint inhibitor treatment. Disease staging was performed according to the 8th edition of the AJCC classification. ECOG, Eastern Cooperative Oncology Group; ULN: up‐per limit of normal; LDH: Lactate dehydrogenase.
**Table S2:** Differentially abundant candidate proteins in the overall resistant group versus the non‐resistant group. Differentially abundant proteins (DEPs) are displayed by regulation status, with Uniprot IDs and gene names. DEPs are displayed with *p* value, fold change, mean value, and standard deviation of the non‐resistant group (group 1) and the resistant group (group 2). Adjusted *p* value indicates the *p* value after Benjamini‐Hochberg correction.
**Table S3:** Metascape pathway cluster enrichment analysis of overall resistance. Metascape pathway enrichment and clustering analysis of proteins associated with overall resistance. Pathways enriched in the overall resistant group compared with the non‐resistant group (*p* < 0.05, FDR < 0.05) are shown. GroupID indicates the pathway cluster, Category the annotation source, Term and Description the enriched pathway, LogP and Log(q‐value) the enrichment significance before and after FDR correction, Symbols the corresponding gene symbols.
**Table S4:** Univariate and multivariable logistic regression for candidate proteins of overall resistance. Logistic regression analysis of DEPs and covariates in the no resistance versus the overall resistance group, displaying the Odds ratio (OR) and confidence interval (CI) of 95%. Significant *p* value at < 0.05. LDH: Lactate dehydrogenase.
**Table S5:** Differentially abundant candidate proteins in the primary resistant group versus no resistance. Differentially abundant proteins (DEPs) are displayed by regulation status, with Uniprot IDs and gene names. DEPs are displayed with *p* value, fold change, mean value, and standard deviation of the non‐resistant group (group 1) and the primary resistant group (group 2). Adjusted *p* value indicates the *p* value after Benjamini‐Hochberg correction.
**Table S6:** Logistic regression analysis of covariates towards primary and secondary resistance. Logistic regression analysis of covariates associated with primary and secondary resistance. Univariate and multivariable logistic regression models were fitted comparing nonresponders to responders only. Odds ratios (ORs) with 95% confidence intervals (CIs) are reported. Due to missing data in covariates, models could not be estimated for all proteins (as of lack of sufficient, i.e., at least 20 cases); in such cases, ORs and CIs are reported as NA. *p* values < 0.05 were considered statistically significant. LDH, lactate dehydrogenase.
**Table S7:** Differentially abundant candidate proteins in the secondary resistant group ver‐sus no resistance. Differentially abundant proteins (DEPs) are displayed by regulation status via Uniprot‐ID and gene name. DEPs are displayed with *p* value, fold change, mean value, and standard deviation of the non‐resistant group (group 1) and secondary resistant group (group 2). Adjusted *p* value indicates the *p* value after Benjamini‐Hochberg correction.
**Table S8:** Multivariable Cox proportional hazards analysis of biomarker signatures and covariates. Multivariable Cox proportional hazards analysis of computed biomarker signatures for overall resistance (left), primary resistance (center), and secondary resistance (right) and covariates. Hazard ratio (HR) indicates the speed of progression, depicted with a confidence interval (CI) of 95%. Significant *p* value at < 0.05. LDH: Lactate dehydrogenase.
**Figure S1:** Transmission electron microscopy of plasma‐derived extracellular vesicles of no resistance and overall resistance group. (A–C) Size and concentration of Extracellular Vesicles (EVs) secreted for (A) no resistance, (B) primary resistance, and (C) secondary resistance conditions, as measured by Nanoparticle tracking analysis (NTA). Histogram showing the calculated mean ± SD of size distribution. Red error bars indicate ±1 standard error of the mean. Screenshots from a video recorded using NanoSight LM14, showing the distribution of EVs. (D, E) Exemplary EV preparations from patients in (D) the no resistance group, and (E) the overall resistance group. Vesicles exhibit the characteristic round to cup‐shaped morphology with a lipid bilayer structure and a size range consistent with small EVs (50–200 nm).
**Figure S2:** Exploratory pathway analysis in primary resistant patients. (A, B) Voronoi diagrams projecting (A) Upregulated biological pathways in the primary resistance group. (B) Downregulated biological pathways in the primary resistance group. (A, B) Colour coding via *p* value, only significant pathways are represented at a cut‐off of *p* < 0.05.
**Figure S3:** Exploratory pathway analysis in secondary versus non‐resistant patients. (A, B) Voronoi diagrams showing (A) upregulated and (B) downregulated biological pathways in the secondary resistance group. (A, B) Colour‐coded by *p* value; only significant pathways are shown at a cut‐off of *p* < 0.05.

## Data Availability

The mass spectrometry proteomics data have been deposited to the ProteomeXchange Consortium via the PRIDE partner repository with the dataset identifier PXD073714. Further data that support the findings of this study are available upon reasonable request from the corresponding author.
