# Supplementary material for: Unraveling the Plasma Extracellular Vesicles Proteome: Predictors of Resistance in Immunotherapy of Advanced Melanoma
Source: Int J Cancer. 2026 Jul 12;159(11):2824–34. doi: 10.1002/ijc.70650 (PMC13637063; doi:10.1002/ijc.70650)
Supplement: Supplementary file 1 [file IJC-159-2824-s001.pdf]

Supplementary Material

Title: Unraveling the Plasma Extracellular Vesicles Proteome: Predictors of Resistance in Immunotherapy of Advanced Melanoma

Authors: Julian Kött, Glenn Geidel, Carmen M.T. Roeper, Lucija Ačkar, Bente Siebels, Hannah Voß, Hartmut Schlüter, Michaela Schweizer, Kilian Müller, Alessandra Rünger, Tim Zell, Benjamin Deitert, Ali Zeinal-Abedini, Isabel Heidrich, Stefan W. Schneider, Klaus Pantel, Daniel J. Smit, Christoffer Gebhardt

Table of contents:

(1) Supplementary Table S1 …………………………………………………………………… page 2
(2) Supplementary Table S2 …………………………………………………………………… page 4
(3) Supplementary Table S3 …………………………………………………………………… page 6
(4) Supplementary Table S4 …………………………………………………………………… page 7
(5) Supplementary Table S5 …………………………………………………………………… page 8
(6) Supplementary Table S6 …………………………………………………………………… page 10
(7) Supplementary Table S7 …………………………………………………………………… page 13
(8) Supplementary Table S8 …………………………………………………………………… page 14
(9) Supplementary Figure S1 ………………………………………………………………… page 15
(10) Supplementary Figure S2 ……………………………………………………………… page 16
(11) Supplementary Figure S3 ……………………………………………………………… page 17

(1) Supplementary Table S1

		Total number of patients	No resistance	Resistance	
				Primary	Secondary
**Total**		46	15	20	11
**Median age at diagnosis (years)**		77.5 (34-101)	78 (35-87)	78 (34-101)	76 (65-89)
**Sex**					
	Male	31	8	16	7
	Female	15	7	4	4
**Type of melanoma**					
	Superficial spreading melanoma	9	3	4	2
	Nodular melanoma	12	2	8	2
	Lentigo maligna melanoma	2	1	0	1
	Acrolentiginous melanoma	1	0	0	1
	Uveal melanoma	4	2	1	1
	Mucosal melanoma	4	1	2	1
	Melanoma of unknown primary	9	2	4	3
	Other	5	4	1	0
**Ulceration**					
	No	17	5	7	5
	Yes	12	4	8	0
	Not applicable/unknown	17	6	5	6
***BRAF* mutated**					
	No	25	7	13	5
	Yes	18	6	7	5
	Unknown	3	2	0	1
**Brain metastasis**					
	Yes	11	5	3	3
	No	35	10	17	8
**AJCC-8 classification (stage)**					
	III C	3	2	1	0
	III D	1	0	1	0
	IV	42	13	18	11
**S100 level (serum, µg/dl)**					
	Normal (≤ ULN)	24	11	10	3
	Elevated (> ULN, 0.150)	20	4	9	7
	Unknown	2	0	1	1
**LDH level (serum, U/l)**					
	Normal (≤ ULN)	17	6	7	4
	Elevated (> ULN, 246)	28	9	12	7
	Unknown	1	0	1	0
**ECOG performance status**					
	0	34	12	12	10
	1	9	3	5	1
	2	3	0	3	0
**Immune checkpoint inhibition**					
	Ipilimumab + Nivolumab	25	11	7	7
	Pembrolizumab	10	1	7	2
	Nivolumab	6	2	3	1
	Other	5	1	3	1
**Treatment line**					
	1	32	11	15	6

	2	10	3	3	4
	> 2	4	1	2	1
**Median time to progress (months)**		8.4	NA	4.7	16.5
**Median follow-up (months)**		27.8	49.5	15.3	34.2

Table S1: Patient characteristics. Clinical characteristics of the advanced melanoma patient cohort showed no resistance, primary resistance, and secondary resistance groups upon immune checkpoint inhibitor treatment. Disease staging was performed according to the 8th edition of the AJCC classification. ECOG, Eastern Cooperative Oncology Group; ULN: up-per limit of normal; LDH: Lactate dehydrogenase.

(2) Supplementary Table S2

uniprot_id	regulation	mean_group1	std_group1	mean_group2	std_group2	p_value	fold_change	abs_logFC	ranking	adjusted_p_value	gene_name
P60953	up_in_resistant	-3.2119417	1.02378397	-1.7053892	1.098035	0.00076108	2.84130265	1.50655251	1	0.227219895	CDC42
Q9UBC9	down_in_resistant	5.27049	0.61043114	-1.2781586	1.05390667	0.00092689	0.01068219	6.5486486	2	0.227219895	SPRR3
P02655	down_in_resistant	3.93384	1.361358	2.23890307	1.82972007	0.00126468	0.30886816	1.69493693	3	0.227219895	APOC2
P01860	up_in_resistant	3.03391707	1.41583678	4.43991057	1.27059725	0.00324903	2.6500021	1.4059935	4	0.37258259	IGHG3
P68104	down_in_resistant	-0.0736419	1.53981881	-2.0617998	1.43091833	0.00345624	0.25206052	1.98815791	5	0.37258259	EEF1A1
P02656	down_in_resistant	6.43187867	1.38568511	5.116754	1.32471711	0.00514129	0.40189077	1.31512467	6	0.461859369	APOC3
P02763	up_in_resistant	-0.6368989	1.45993681	0.82528722	1.85172576	0.00663623	2.75525554	1.46218613	7	0.510989987	ORM1
P62805	down_in_resistant	1.02512731	1.61378574	-0.3848262	1.46123809	0.00828302	0.37632382	1.40995349	8	0.558068421	HIST1H4A
P02768	up_in_resistant	9.00094867	1.3954493	10.2246626	1.50724645	0.01090167	2.33547162	1.22371391	9	0.581141757	ALB
Q01518	up_in_resistant	-3.03198	0.51650017	-0.6404816	2.21256363	0.01246273	5.24702057	2.39149844	10	0.581141757	CAP1
P02774	up_in_resistant	-1.0889641	2.71871272	1.07511707	2.21115268	0.01402063	4.48180889	2.16408113	11	0.581141757	GC
P02790	up_in_resistant	0.99436529	1.91087492	2.60595722	2.13997051	0.01550902	3.05588854	1.61159192	12	0.581141757	HPX
A0A0B4J1Y8	down_in_resistant	-0.1931078	0.37572571	-1.676885	1.14216791	0.01559975	0.35755146	1.4837772	13	0.581141757	IGLV9-49
P0DOX5	up_in_resistant	5.47232867	1.40729125	6.59730548	1.54866846	0.019873	2.18098042	1.12497682	16	0.581141757	IGKC
P15153	up_in_resistant	-1.8500933	1.14420486	-0.7535724	1.46731117	0.02155658	2.13838386	1.09652085	19	0.581141757	RAC2
Q9NQ84	down_in_resistant	-1.2721203	1.19064206	-3.551285	0.75809517	0.02264189	0.20601699	2.27916475	21	0.581141757	GPRC5C
P29401	down_in_resistant	-2.364238	0.88054656	-3.8175867	1.17197062	0.02447138	0.36517283	1.45334867	22	0.599548902	TKT
P02654	down_in_resistant	5.06953333	1.48560221	3.99381513	1.45062036	0.02809093	0.47443482	1.0757182	23	0.658304865	APOC1
P01704	up_in_resistant	-3.3778255	3.45956191	-0.0976692	1.11237486	0.03187602	9.71461139	3.28015629	25	0.658708481	IGKV3-20
Q13885	up_in_resistant	-2.29697	1.00471768	0.37323465	0.58373977	0.03310489	6.36519473	2.67020465	26	0.658708481	TUBB2A
P00746	down_in_resistant	0.13329053	0.93657467	-1.5338703	1.29808263	0.03860927	0.3148724	1.66716081	28	0.658708481	CFD
P22531	up_in_resistant	-3.09314	0.54507167	-1.8020938	1.46429244	0.03870048	2.44705441	1.29104618	29	0.658708481	SPRR2E
A0A0B4J1X8	down_in_resistant	0.4351866	0.53403023	-1.7428867	0.4957411	0.03927063	0.22097066	2.17807327	30	0.658708481	IGHV3-43
P01859	up_in_resistant	2.80575353	1.87887947	4.07950252	1.86773098	0.03963467	2.41789062	1.27374898	31	0.658708481	IGHG2
P61626	down_in_resistant	2.56715253	1.5508614	1.55264716	1.34701313	0.04001993	0.49499801	1.01450537	32	0.658708481	LYZ
A0M8Q6	up_in_resistant	-4.29265	0.4309533	-2.8806483	1.55508034	0.04285481	2.66106122	1.4120017	33	0.658708481	IGLC7
Q08431	up_in_resistant	-3.50616	0.61699854	-2.2007894	1.0316627	0.0451095	2.47147202	1.30537057	34	0.658708481	MFGE8
Q96DA0	down_in_resistant	-1.9289674	1.76982401	-3.295887	1.46831984	0.04563893	0.3877182	1.36691963	35	0.658708481	ZG16B

P02751	up_in_resistant	-0.5984978	2.49255648	1.08652471	1.61743077	0.04941219	3.21545402	1.68502246	36	0.658708481	FN1

Table S2: Differentially abundant candidate proteins in the overall resistant group versus the non-resistant group. Differentially abundant proteins (DEPs) are displayed by regulation status, with Uniprot IDs and gene names. DEPs are displayed with p-value, fold change, mean value, and standard deviation of the non-resistant group (group 1) and the resistant group (group 2). Adjusted p-value indicates the p-value after Benjamini-Hochberg correction.

(3) Supplementary Table S3

GroupID	Category	Term	Description	LogP	Log(q-value)	Symbols
1_Summary	Reactome Gene Sets	R-HSA-76002	Platelet activation, signaling and aggregation	-9.7495	-5.407164	ALB,CDC42,FN1,ORM1,RAC2,CAP1,TUBB2A
2_Summary	Reactome Gene Sets	R-HSA-8957275	Post-translational protein phosphorylation	-5.1451	-1.501701	ALB,FN1,MFGE8,CDC42
3_Summary	KEGG Pathway	hsa05135	Yersinia infection	-4.8256	-1.358422	CDC42,FN1,RAC2,CAP1,TUBB2A
4_Summary	GO Biological Processes	GO:0001667	ameboidal-type cell migration	-4.7977	-1.358422	CDC42,FN1,CAP1,MFGE8,TUBB2A
5_Summary	Reactome Gene Sets	R-HSA-5653656	Vesicle-mediated transport	-2.7982	0	ALB,HPX,TUBB2A

Table S3: Metascape pathway cluster enrichment analysis of overall resistance. Metascape pathway enrichment and clustering analysis of proteins associated with overall resistance. Pathways enriched in the overall resistant group compared with the non-resistant group ($p < 0.05$, FDR < 0.05) are shown. *GroupID* indicates the pathway cluster, *Category* the annotation source, *Term* and *Description* the enriched pathway, *LogP* and *Log(q-value)* the enrichment significance before and after FDR correction, *Symbols* the corresponding gene symbols.

(4) Supplementary Table S4

	univariable		multivariable	
	OR (CI)	p-value	OR (CI)	p-value
IGHG3 (high / low)	2.23 (1.33-4.29)	0.006	2.43 (1.14-5.15)	0.021
APOC2 (high / low)	0.45 (0.23-0.75)	0.007	0.30 (0.13-0.71)	0.007
APOC3 (high/low)	0.46 (0.23-0.78)	0.010	0.16 (0.04-0.69)	0.024
HIST1H4A (high / low)	0.51 (0.28-0.82	0.012	0.43 (0.19-0.98)	0.045
GC (high / low)	1.44 (1.10-1.99)	0.013	1.24 (0.85-1.79)	0.261
ORM1 (high / low)	1.63 (1.12-2.60)	0.020	1.56 (0.86-2.81)	0.141
ALB (high / low)	1.73 (1.12-2.89)	0.021	1.45 (0.82-2.58)	0.200
HPX (high / low)	1.46 (1.07-2.12)	0.026	1.24 (0.85-1.79)	0.322
IGKC (high / low)	1.69 (1.10-2.84)	0.026	2.03 (1.05-3.91)	0.034
APOC1 (high / low)	0.56 (0.31-0.90)	0.028	0.46 (0.22-0.93)	0.032
SERPINA1 (high / low)	1.77 (1.04-3.33)	0.051		
S100 (elevated / normal)	1.16 (0.84-1.93)	0.450		
LDH (elevated / normal)	1.00 (0.99-1.01)	0.120		
sex (female / male)	0.34 (0.09-1.30)	0.117		
BRAF (mutated / not mutated)	0.82 (0.22-3.15)	0.773		
Age (high / low)	1.04 (0.99-1.10))	0.111		

Table S4: Univariate and multivariable logistic regression for candidate proteins of overall resistance. Logistic regression analysis of DEPs and covariates in the no resistance versus the overall resistance group, displaying the Odds ratio (OR) and confidence interval (CI) of 95 %. Significant p-value at < 0.05. LDH: Lactate dehydrogenase.

(5) Supplementary Table S5

uniprot_id	regulation	mean_group1	std_group1	mean_group2	std_group2	p_value	fold_change	abs_logFC	ranking	adjusted_p_value	gene_name
P68104	down_in_resistant	-0.0736419	1.53981881	-2.6947189	1.306996676	0.00069915	0.16254634	2.621077	1	0.282719826	EEF1A1
P60953	up_in_resistant	-3.21194167	1.02378397	-1.62188271	1.001136519	0.00114829	3.01061653	1.59005896	2	0.282719826	CDC42
P62805	down_in_resistant	1.025127313	1.61378574	-0.70263862	1.251924732	0.00199201	0.30191913	1.72776593	3	0.282719826	HIST1H4A
P02763	up_in_resistant	-0.63689891	1.45993681	0.954612185	1.369551689	0.00270941	3.01364836	1.59151109	4	0.282719826	ORM1
P02768	up_in_resistant	9.000948667	1.3954493	10.4421795	1.152371868	0.00307554	2.71552441	1.44123083	5	0.282719826	ALB
P01860	up_in_resistant	3.033917067	1.41583678	4.4347005	1.010306437	0.00330667	2.64044929	1.40078343	6	0.282719826	IGHG3
P02774	up_in_resistant	-1.08896406	2.71871272	1.367196105	1.66821396	0.0055688	5.48754234	2.45616017	7	0.390760692	GC
P16402	down_in_resistant	-2.16662729	1.38390622	-4.31739667	0.421488589	0.00609373	0.22519249	2.15076938	8	0.390760692	HIST1H1D
P0DOX5	up_in_resistant	5.472328667	1.40729125	6.706549	1.087729938	0.00909214	2.35254176	1.23422033	9	0.518252005	IGKC
P02790	up_in_resistant	0.994365293	1.91087492	2.74133535	1.824599633	0.0106048	3.35652888	1.74697006	10	0.544026143	HPX
Q01469	down_in_resistant	1.151391314	1.19238962	0.119657135	1.096993058	0.01627214	0.48912185	1.03173418	11	0.59360605	FABP5
P06733	down_in_resistant	0.55217602	1.62738486	-1.19799309	1.646773514	0.01650819	0.29726693	1.75016911	12	0.59360605	ENO1
P02655	down_in_resistant	3.93384	1.361358	2.57569085	1.865838994	0.01800855	0.39008241	1.35814915	13	0.59360605	APOC2
P61626	down_in_resistant	2.567152533	1.5508614	1.3222794	1.389331591	0.02041484	0.421945	1.24487313	14	0.59360605	LYZ
P68431	down_in_resistant	-0.1652604	1.29029472	-1.20179453	0.998169751	0.02072032	0.48749721	1.03653413	15	0.59360605	HIST1H3A
P01704	up_in_resistant	-3.3778255	3.45956191	0.144117333	0.60023485	0.02365415	11.4871009	3.52194283	16	0.59360605	IGKV3-20
A0A0B4J1Y8	down_in_resistant	-0.1931078	0.37572571	-1.80657157	1.281200556	0.02392091	0.32681276	1.61346377	17	0.59360605	IGLV9-49
P01859	up_in_resistant	2.805753533	1.87887947	4.1777325	1.40442068	0.02559814	2.58825357	1.37197897	20	0.59360605	IGHG2
P04792	down_in_resistant	-0.02450755	1.77277044	-1.69021361	1.602032048	0.02660854	0.31519006	1.66570606	22	0.59360605	HSPB1
P02787	up_in_resistant	4.290582	1.72728404	5.650516	1.726640804	0.02816296	2.56673437	1.359934	24	0.601983344	TF
Q9NQ84	down_in_resistant	-1.27212025	1.19064206	-3.47644667	0.910196554	0.03934395	0.21698596	2.20432642	26	0.698418049	GPRC5C
Q01518	up_in_resistant	-3.03198	0.51650017	-0.165916	1.753739478	0.04176217	7.29073367	2.866064	27	0.698418049	CAP1
A0A0G2JS06	down_in_resistant	-1.27219175	0.60050147	-2.67938	1.347786257	0.04192093	0.37704582	1.40718825	28	0.698418049	IGHV
Q16378	down_in_resistant	0.545247395	1.53643718	-0.59417484	1.542464635	0.04537058	0.45394133	1.13942224	29	0.698418049	PRR4

Q96DA0	down_in_resistant	-1.92896737	1.76982401	-3.4638578	1.60516863	0.0457807	0.34510555	1.53489043	30	0.698418049	ZG16B
P21730	up_in_resistant	-3.53432	0.68226883	-2.1538225	0.575903678	0.0479964	2.60358138	1.3804975	32	0.698418049	C5AR1

Table S5: Differentially abundant candidate proteins in the primary resistant group versus no resistance. Differentially abundant proteins (DEPs) are displayed by regulation status, with Uniprot IDs and gene names. DEPs are displayed with p-value, fold change, mean value, and standard deviation of the non-resistant group (group 1) and the primary resistant group (group 2). Adjusted p-value indicates the p-value after Benjamini-Hochberg correction.

(6) Supplementary Table S6

	primary resistance univariate		primary resistance multivariate		secondary resistance univariate		secondary resistance multivariate	
	OR (CI)	p-value	OR (CI)	p-value	OR (CI)	p-value	OR (CI)	p-value
IGHG3 (high / low)	**2.81 (1.44-7.23)**	**0.001**	**4.19 (1.22-14.37)**	**0.022**				
GC (high / low)	**1.65 (1.18-2.53)**	**0.001**	1.49 (0.91-2.44)	0.113				
HIST1H4A (high / low)	**0.31 (0.11-0.64)**	**0.001**	0.24 (0.05-1.09)	0.064				
ALB (high / low)	**2.31 (1.31-4.71)**	**0.009**	1.68 (0.84-3.36)	0.143				
ORM1 (high / low)	**2.34 (1.33-4.98)**	**0.010**	2.64 (0.99-7.06)	0.052				
IGKC (high / low)	**2.63 (1.36-6.42)**	**0.012**	**6.00 (1.30-27.71)**	**0.022**				
HPX (high / low)	**1.65 (1.13-2.63)**	**0.018**	1.44 (0.85-2.44)	0.172				
ENO1 (high / low)	**0.45 (0.19-0.81)**	**0.023**	0.26 (0.06-1.19(	0.082				
HIST1H3A(high / low)	**0.39 (0.15-0.81)**	**0.024**	0.08 (0.01-1.02)	0.052				
LYZ (high / low)	**0.53 (0.28-0.88)**	**0.027**	0.58 (0.30-1.12)	0.106				
IGHG2 (high / low)	**1.75 (1.11-3.13)**	**0.030**	2.66 (1.00-7.07)	0.050				
APOC2 (high / low)	**0.55 (0.29-0.89)**	**0.034**	**0.38 (0.16-0.89)**	**0.027**				
FABP5 (high / low)	**0.45 (0.19-0.88)**	**0.036**	0.25 (0.06-1.05)	0.059				
TF(high / low)	**1.56 (1.04-2.56)**	**0.046**	1.28 (0.76-2.16)	0.344				
HSPB1 (high / low)	**0.57 (0.30-0.94)**	**0.046**	0.24 (0.03-1.88)	0.172				
**APOC1** (high / low)					**0.23 (0.05-0.59)**	**0.017**	**0.14 (0.02-0.97)**	**0.047**

APOC3 (high / low)					**0.22 (0.05-0.59)**	**0.018**	0.04 (0.01-2.33)	0.123
Q86UX7 (high / low)					**2.45 (1.26-6.64)**	**0.025**	NA	
FERMT3 (high / low)					**2.32 (1.11-5.95)**	**0.042**	NA	
PF4 (high / low)					**0.54 (0.26-0.92)**	**0.044**	0.26 (0.06-1.09)	0.066
GNB1 (high / low)					**2.03 (1.10-4.79)**	**0.048**	NA	
C4B (high / low)					**1.66 (1.06-3.03)**	**0.049**	NA	
APOE (high / low)					0.54 (0.28-0.96)	0.051		
CDC42 (high / low)					3.41 (1.24-18.11)	0.057		
S100A9 (high / low)					0.51 (0.23-0.95)	0.059		
SERPING1 (high / low)					1.79 (1.02-3.89)	0.073		
S100 (elevated / normal)	1.21 (0.88-1.98)	0.310			1.00 (0.99-1.01)	0.849		
LDH (elevated / normal)	1.00 (0.99-1.01)	0.090			1.00 (0.99-1.21)	0.268		
sex (female / male)	2.14 (0.04-0.99)	0.059			0.65 (0.12-3.17)	0.600		
BRAF (mutated / not mutated)	0.68 (0.16-2.89)	0.599			1.17 (0.22-6.28)	0.855		
Age (high / low)	1.04 (0.99-1,10)	0.137			1.03 (0.97-1.11)	0.310		

Table S6: Logistic regression analysis of covariates towards primary and secondary resistance. Logistic regression analysis of covariates associated with primary and secondary resistance. Univariate and multivariable logistic regression models were fitted comparing non-responders to responders only. Odds ratios (ORs) with 95% confidence intervals (CIs) are reported. Due to missing data in covariates, models could not be estimated for all proteins (as of lack of sufficient, i.e. at least 20 cases); in such cases, ORs and CIs are reported as NA. P values < 0.05 were considered statistically significant. LDH, lactate dehydrogenase.

(7) Supplementary Table S7

uniprot_id	regulation	mean_group1	std_group1	mean_group2	std_group2	p_value	fold_change	abs_logFC	ranking	adjusted_p_value	gene_name
P02656	down_in_resistant	6.431878667	1.38568511	4.287716	1.19381237	0.000471	0.22622611	2.14416267	1	0.243508832	APOC3
P02654	down_in_resistant	5.069533333	1.48560221	2.88647446	1.41940282	0.00096877	0.22020836	2.18305888	2	0.250427033	APOC1
P02655	down_in_resistant	3.93384	1.361358	1.5653275	1.63765013	0.00149454	0.19364518	2.3685125	3	0.257559077	APOC2
Q86UX7	up_in_resistant	-0.8345965	1.64335276	1.286792	1.31823055	0.00528918	4.35112511	2.1213885	4	0.68362584	FERMT3
Q9UBC9	down_in_resistant	5.27049	0.61043114	-1.7075575	0.50173326	0.00891463	0.00793229	6.9780475	5	0.921772709	SPRR3
P62873	up_in_resistant	-2.60100217	1.6844271	-0.86440863	1.46733689	0.02613251	3.33247382	1.73659354	6	0.980475054	GNB1
P02776	down_in_resistant	2.085328286	1.67246851	0.32637256	1.74752727	0.02864956	0.29546195	1.75895573	7	0.980475054	PF4
Q99436	up_in_resistant	-3.90264	0.22701671	-1.943435	0.3379334	0.03221706	3.88847645	1.959205	8	0.980475054	PSMB7
P0C0L5	up_in_resistant	1.328021462	2.12975005	3.46114111	2.1218523	0.03306692	4.38665015	2.13311965	9	0.980475054	C4B
P60953	up_in_resistant	-3.21194167	1.02378397	-1.83661357	1.30872621	0.03742277	2.59426903	1.3753281	10	0.980475054	CDC42
P15153	up_in_resistant	-1.85009325	1.14420486	-0.50279178	1.37355405	0.03883795	2.54435764	1.34730148	11	0.980475054	RAC2
P61769	up_in_resistant	-2.66437673	1.40232984	-1.40102829	1.0127663	0.04199233	2.40052247	1.26334844	12	0.980475054	B2M
P05154	up_in_resistant	-3.718604	1.3677894	-0.62873525	1.96649132	0.04281541	8.51418684	3.08986875	13	0.980475054	SERPINA5
P06702	down_in_resistant	3.726132	1.39102124	2.55185082	1.36885737	0.0431839	0.44310448	1.17428118	15	0.980475054	S100A9
P05155	up_in_resistant	-0.93346246	1.9373474	0.75928263	1.46496977	0.04466844	3.23271221	1.69274508	16	0.980475054	SERPING1
P02649	down_in_resistant	7.943578	1.36720478	6.68915909	1.55787902	0.04536771	0.41916236	1.25441891	17	0.980475054	APOE
P55058	up_in_resistant	-2.9095488	2.17569387	-0.1993835	0.63233324	0.04719815	6.54396622	2.7101653	18	0.980475054	PLTP
P01860	up_in_resistant	3.033917067	1.41583678	4.4503307	1.74555985	0.04760046	2.66921152	1.41641363	19	0.980475054	IGHG3

Table S7: Differentially abundant candidate proteins in the secondary resistant group versus no resistance. Differentially abundant proteins (DEPs) are displayed by regulation status via Uniprot-ID and gene name. DEPs are displayed with p-value, fold change, mean value, and standard deviation of the non-resistant group (group 1) and secondary resistant group (group 2). Adjusted p-value indicates the p-value after Benjamini-Hochberg correction.

(8) Supplementary Table S8

	overall resistance		primary resistance		secondary resistance	
	HR (CI)	p-value	HR (CI)	p-value	HR (CI)	p-value
signature	4.39 (1.73-11.14)	0.002	5.74 (1.66-19.84)	0.005	8.76 (1.37-55.66)	0.021
LDH (per ten units increase)	1.06 (1.02-1.10)	0.002	1.08 (1.03-1.13)	0.001	1.08 (0.97-1.20)	0.141
BRAF (mutated / not mutated)	0.32 (0.12-0.85)	0.022	0.32 (0.07-1.36)	0.122	0.39 (0.08-1.93)	0.251
age (per year)	1.04 (0.99-1.09)	0.135	1.05 (0.98-1.15)	0.151	1.02 (0.93-1.11)	0.678
sex (female/male)	0.65 (0.24-1.80)	0.408	0.40 (0.09-1.75)	0.223	0.62 (0.12-3.17)	0.569
S100 (per unit increase)	1.00 (0.76-1.33)	0.980	0.95 (0.69-1.31)	0.760	0.65 (0.29-1.48)	0.302

Table S8: Multivariable Cox proportional hazards analysis of biomarker signatures and covariates. Multivariable Cox proportional hazards analysis of computed biomarker signatures for overall resistance (left), primary resistance (center), and secondary resistance (right) and covariates. Hazard ratio (HR) indicates the speed of progression, depicted with a confidence interval (CI) of 95 %. Significant p-value at < 0.05. LDH: Lactate dehydrogenase.

(9) Supplementary Figure S1

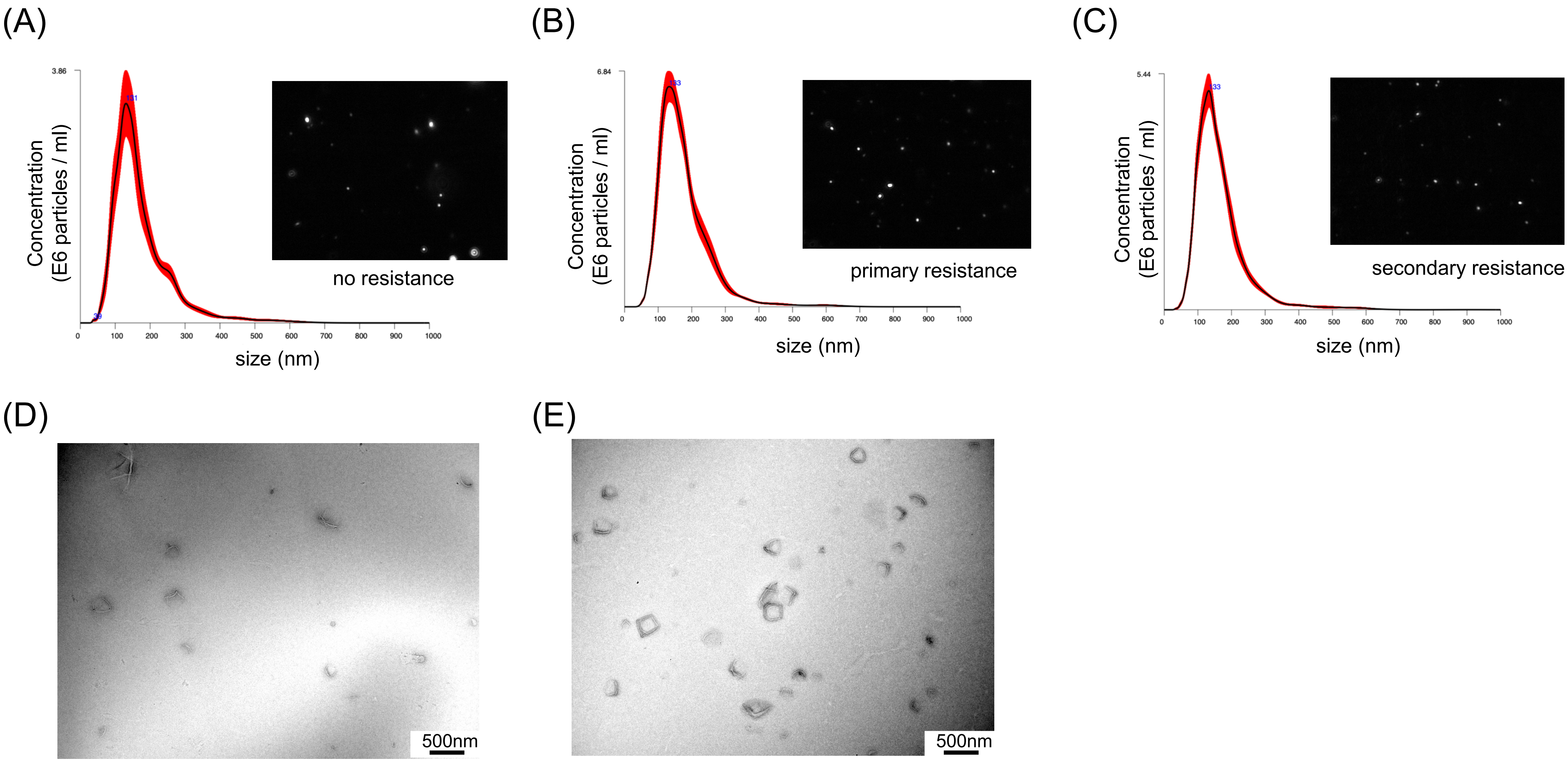

Transmission electron microscopy of plasma-derived extracellular vesicles of no resistance and overall resistance group. (A-C) Size and concentration of Extracellular Vesicles (EVs) secreted for (A) no resistance, (B) primary resistance, and (C) secondary resistance conditions, as measured by Nanoparticle tracking analysis (NTA). Histogram showing the calculated mean ± SD of size distribution. Red error bars indicate ± 1 standard error of the mean. Screenshots from a video recorded using NanoSight LM14, showing the distribution of EVs. (D-E) Exemplary EV preparations from patients in (D) the no resistance group, and (E) the overall resistance group. Vesicles exhibit the characteristic round to cup-shaped morphology with a lipid bilayer structure and a size range consistent with small EVs (50–200 nm).

(10) Supplementary Figure S2

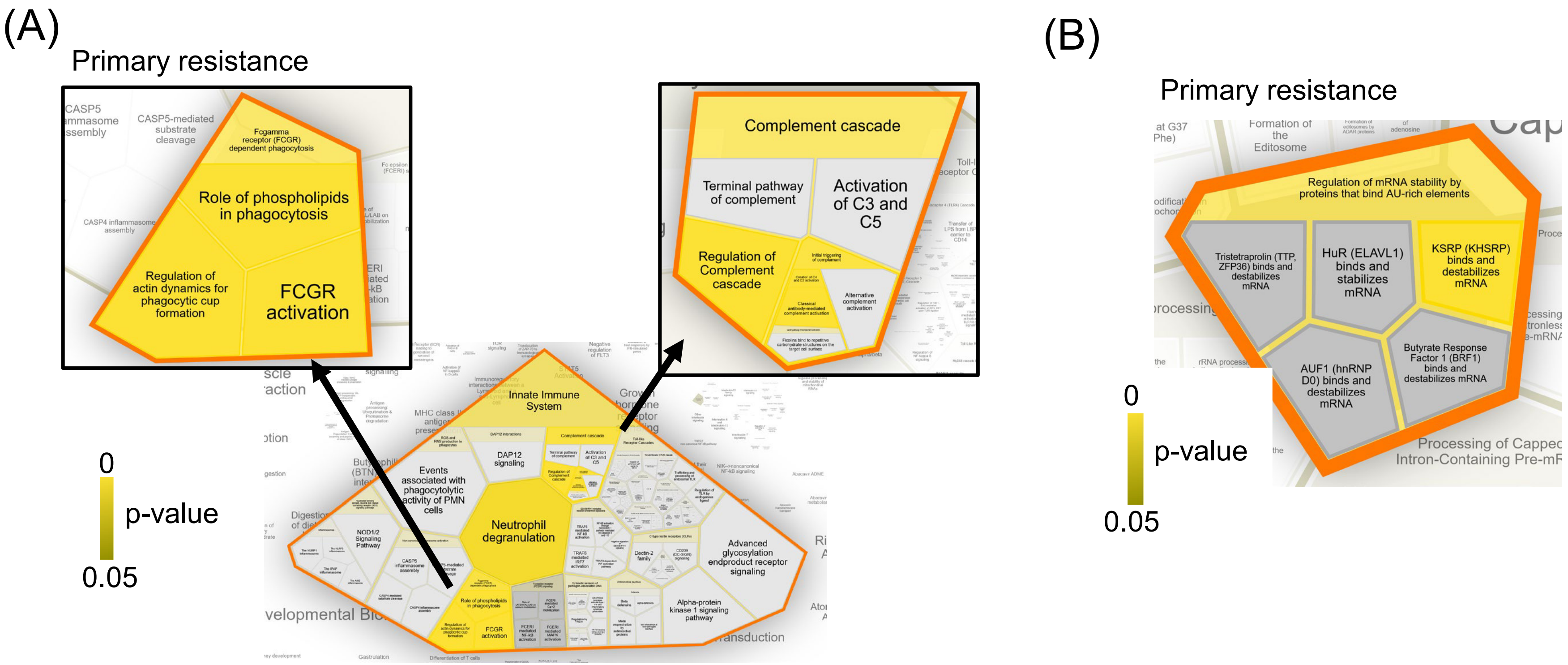

Exploratory pathway analysis in primary resistant patients. (A,B) Voronoi diagrams projecting (A) Upregulated biological pathways in the primary resistance group. (B) Downregulated biological pathways in the primary resistance group. (A,B) Colour coding via p-value, only significant pathways are represented at a cut-off of $p < 0.05$.

(11) Supplementary Figure S3

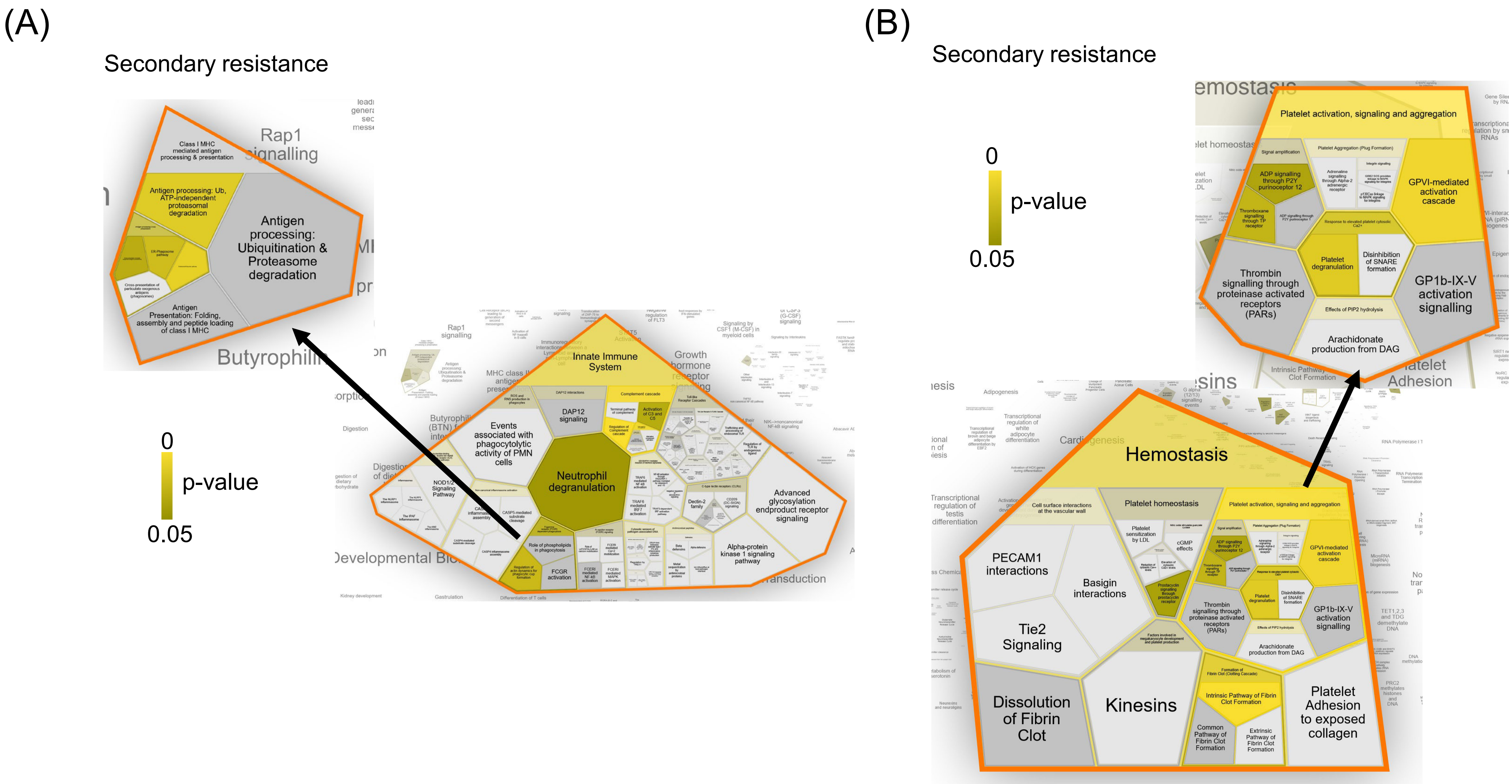

Exploratory pathway analysis in secondary versus non-resistant patients. (A, B) Voronoi diagrams showing (A) upregulated and (B) downregulated biological pathways in the secondary resistance group. (A,B) Colour-coded by p-value; only significant pathways are shown at a cut-off of $p < 0.05$.
